# Editorial: The Variable Mind? How Apparently Inconsistent Effects Might Inform Model Building

**DOI:** 10.3389/fpsyg.2016.00185

**Published:** 2016-02-22

**Authors:** Simona Amenta, Davide Crepaldi

**Affiliations:** ^1^Center for Mind/Brain Sciences, University of TrentoRovereto, Italy; ^2^International School for Advanced StudiesTrieste, Italy; ^3^Milan Center for NeuroscienceMilan, Italy

**Keywords:** experimental variability, individual differences, reading, bilingualism, dyslexia

Human behavior is very difficult to predict precisely, as even the exact same cognitive system may respond differently to very similar input. That is, the study of experimental psychology and neuroscience requires dealing with a huge amount of variability. Our response to this state of affairs, as a field, has been dominated by the (rather tacit) assumption that variability means noise, and thus it is something we need to (i) ignore theoretically; and (ii) fight against experimentally, searching for stable effects. Although it remains obvious that part of the variability we see in our experiments is indeed noise, a different approach emerged recently, based on the assumption that the cognitive system is guided by dynamic and flexible architectures that adapt quickly to different contexts. Thus, how psychological effects emerge and disappear in different, e.g., people, contexts, languages, brings light into the features of the cognitive system itself (e.g., Norris, 2006; Andrews and Lo, 2013). Variability is in focus as an intrinsic aspect of cognitive processing, rather than a sign of experimental weakness; and the experimental and theoretical enterprise is directed toward the validation of consistently variable facts. The present E-book is a collection of experimental and theoretical work that moves in this direction, focusing on how variability may inform theoretical advance.

The focus on variability has been interpreted in terms of context effects by Danelli et al. and Monsalve et al. The former group conducted an fMRI experiment where similar sets of regular words were presented to participants together with either nonwords or irregular words, in an attempt to enhance grapheme–to–phoneme or lexical–semantic reading respectively, in the context of dual–route models of reading (e.g., Coltheart et al., 2001). Brain activations were partially different in the two contexts, thus leading Danelli et al. to claim association between different neural circuits and either sub–lexical or lexical reading. Monsalve et al. compared ERPs elicited by the same words when embedded in: (i) multiword expressions (e.g., “kick the bucket”); (ii) highly predictable, but non–fixed compositional structures (e.g., “the opposite of black is white”); or (iii) non–constraining contexts (e.g., “Phil asked Mary to bring her ring”). The same exact set of words brought about different neural responses in different contexts, in this case teasing apart lexical identification and word prediction.

Variability across languages is another hot issue in this Research Topic. Focusing on English and French, Casalis et al. assessed the role of morphemes in the reading performance of a group of children. In both languages, the presence of derivational morphemes facilitates word recognition (e.g., “postal” better than “turnip”), and hinders nonword rejection (e.g., “pondal” worse than “curlip”). However, the same factor affects latencies in the two languages in different ways, possibly due to the different derivational structures of English and French.

Differences between language orthographies are explored in the study by Marinelli et al., who compare reading performance of English and Italian young adults in a series of three experiments. By means of an ex–Gaussian distribution analysis, the authors unveiled more diversity among English readers in terms of response time variability and the amount of slow responses; but not in terms of mean, which is what goes under the microscope more often. Traficante and Burani contributed a review that focuses on how different orthographic systems (particularly in terms of letter–to–sound mapping consistency) shape different ways in which the lexical and sub–lexical reading routes are (de)emphasized according to context. Yum et al. built on the special features of Chinese to tease apart regularity (how much the pronunciation of a word follows letter–to–sound conversion rules) and consistency (how much the pronunciation of a word is consistent with that of words with similar orthography). In a series of ERP studies, the authors find indeed different timings and different directions for the electrophysiological effects of the two constructs.

Dellantonio et al. focused instead on differences between types of words within the same language. Building on the MRC database (Coltheart, 1981), they were able to identify groups of words where the classic correlation between imageability and concreteness ratings doesn't hold, thus helping clarifying the difference between the two constructs.

Inter–individual variability is the target in Robidoux and Pritchard, who compared the responses predicted by the DRC (Coltheart et al., 2001) and the CDP++ (Perry et al., 2010) models of reading to those of a group of human subjects. By means of hierarchical clustering, they individuated groups of subjects that differ in the pronunciation of specific consonant clusters. Based on this finding, Robidoux and Pritchard compared DRC and CDP++ for their ability to model different, but internally consistent, reading profiles, setting a new and interesting way to address the long–lasting issue of adjudicating between different reading models.

Individual differences obviously go well beyond different mappings between sounds and graphemes in fully functional adults. Szterman and Friedmann, for example, analyzed the difficulties that children with impaired hearing show with Wh-movement sentences, highlighting differences in syntactic processing mainly related to the use of a hearing device within the first year of life. Uccula et al. focused instead on the Meares–Irlen syndrome, a condition whereby readers experience eyestrain and/or visual distortions, and reading improves quite dramatically through the use of colored overlays applied above written text.

Finally, the ability to speak more than one language has been quite consistently linked to the ability to outperform monolinguals in a variety of tasks (e.g., Bialystok, 2001). However, by adopting new Bayesian analyses on a large sample, Antón et al. were able to question this connection showing that, in a series of tasks, performance of bilinguals and monolinguals is statistically undistinguishable.

Overall, we are confident that this Research Topic provides solid examples of how consistent variability can inform psychological theory. Contributions cover diverse populations, as well as diverse techniques, which proves that language psychology is indeed widening its toolbox by including the study of how phenomena vary across tasks, contexts, languages, words and people. We hope that this move, still in its early phase, will consolidate and provide more and more insight into the beauty of the human cognitive system.

## Author contributions

SA and DC equally shared the task of editing the content of this Research Topic, and the authorship of this Editorial.

## Funding

This work was funded by a “FIRB-Futuro in Ricerca” grant awarded to DC by the Italian Ministry of Education, University and Research (RBFR085K98).

### Conflict of interest statement

The authors declare that the research was conducted in the absence of any commercial or financial relationships that could be construed as a potential conflict of interest.
